# Playing for Keeps: Long‐Term Recall With an Application Using Virtual Reality and the Method of Loci

**DOI:** 10.1111/sjop.70089

**Published:** 2026-03-23

**Authors:** Josefin Hagström, Anders Winman

**Affiliations:** ^1^ Department of Psychology Uppsala University Uppsala Sweden; ^2^ Department of Women's and Children's Health Uppsala University Uppsala Sweden

**Keywords:** method of loci, mnemonic techniques, motivation, spatial memory, virtual reality

## Abstract

Strategies that provide spatial context to study material have been successfully utilized combined with virtual 3D technology to improve memory and learning. However, research suggests that memory enhancement effects relying on visualization may be of a short‐term nature. The current study aimed to test whether these effects persist after a retention interval of 3 weeks. Additionally, we explored correlations with perceived efficiency, motivation, and gaming experience. German nouns with gender assignment were presented to 48 participants, either without or within a provided spatial context by a virtual reality application. Experimental effects persisted over a three‐week retention interval and were unrelated to motivation and gaming experience. These effects were pronounced, with a 50% memory boost in the experimental condition and increased over time, suggesting decreased forgetting rate. Findings provide insights on the potential of learning applications based on spatial memory and individual factors.

## Key Points

- Using an experimental approach, this study compared a virtual reality–supported Method of Loci (MOL) with traditional text‐based learning for acquiring German grammatical gender.
- Recall performance was assessed using cued and free recall immediately after learning and after a three‐week retention interval.
- The VR‐supported MOL condition produced higher recall accuracy and a shallower forgetting curve than the control condition.
- Individual differences (motivation, perceived efficiency, gaming experience, and biological gender) predicted performance in the control condition but not in the VR‐supported MOL condition.
- These findings suggest that spatially structured, imagery‐based learning environments can support durable associative memory while reducing reliance on learner‐specific factors.

## 1 Introduction

Rapid technological developments during recent decades have brought learners easier access to knowledge and provided new opportunities to apply cognitive psychological findings to educational practice. One promising avenue is the integration of visual imagery into virtual learning environments, leveraging the evolutionary connection between spatial navigation and memory.

### 1.1 Spatial Memory, Method of Loci, and Virtual Reality

Episodic memory, a component of long‐term memory, enables encoding and retrieval of information connected to time and space (Tulving 2002). The Method of Loci (MOL), or the memory palace technique, is a well‐known mnemonic that harnesses visuo‐spatial imagery by associating to‐be‐remembered items with specific spatial locations (Legge et al. 2012). This reliance on spatial context is thought to reflect an evolutionarily based memory mechanism, supported by neural systems involved in navigation, including place cells in the hippocampus and grid cells in the medial temporal lobe (Miller et al. 2013). A systematic review including 83 studies found strong evidence for a large effect on immediate serial recall compared with rehearsal (Ondřej 2025).

Virtual reality (VR) technology provides computer‐generated three‐dimensional (3D) environments that allow users to navigate and interact in spatially structured contexts. According to the dual‐coding theory (Paivio 2014), memory is strengthened when verbal material is encoded alongside imagery‐based representations. Implementing MOL in VR may further enhance these mechanisms, as immersive spatial and visual cues create richer associative networks than imagined loci alone. In previous work, we found that a VR‐based MOL approach enhanced memory for German grammatical gender compared to text‐based learning, with benefits observed at retention intervals of 30 min and 1 week (Hagström and Winman 2018). While similar approaches applying non‐immersive VR have produced weaker effects for vocabulary memorization (McLeod 2007), this may be due to differences in item presentation, as the latter study presented items only as text rather than with images.

### 1.2 Linguistic Gender

Linguistic gender has been ranked among the most complex linguistic categories in second language acquisition. In German, there are three genders: Feminine, masculine and neuter, marked with the determiners *die*, *der* and *das*. Gender assignment remains a persistent source of error even for advanced learners (Rogers 1987), because it is often arbitrary and not transparently encoded in semantic meaning nor morphological form. This arbitrariness makes grammatical genders suitable for studying memory‐based learning, since acquisition relies on associations between nouns and determiners rather than predictable rules. Such associations can then be mapped onto the item‐location connections that are central to MOL. Meanwhile, Swedish employs only two types of gender: *common* and *neuter*, where neither is related to the concept of biological gender.

### 1.3 Potential Moderators

It is possible that several factors may moderate the effects of VR‐supported mnemonic learning. Prior research suggests that motivation and perceived usefulness may influence performance (Hagström and Winman 2018; Huttner et al. 2019), as learners may engage more with methods they perceive as more effective or enjoyable. Furthermore, previous gaming has been correlated with improved performance in VR spatial tasks, potentially reflecting greater familiarity with navigation and interaction in digital environments (Richardson and Collaer 2011). In contrast to non‐gamers, frequent gamers have been found to experience a greater game ease of use (Warden et al. 2016). Lastly, biological gender has been discussed as a possible moderator, given reported differences in gaming experience and spatial navigations skills, although research remains inconclusive (Astur et al. 2016; Chamizo et al. 2011). Therefore, these variables were considered as potential moderators in the present study.

### 1.4 Aims

A literature review (Huang et al. 2021) observed that most studies on VR in language education have focused on vocabulary acquisition, with few examining grammar learning. Furthermore, no studies have retention beyond 1 week. This is essential as some research has suggested that imagery‐based mnemonic techniques, while effective in the short term, may produce rapid forgetting over longer intervals (Dunlosky et al. 2013).

Therefore, this study aims to test long‐term learning in virtual environments by embedding the to‐be‐remembered material in a spatiotemporal context. The main hypothesis is that the memory benefits afforded by VR‐supported MOL will persist over a three‐week retention interval, providing insight into the durability of spatial mnemonic learning. Secondary aims include exploring potential moderators of performance, such as motivation, perceived efficiency, computer gaming experience, and biological gender, as well as the influence of time spent on each task.

## 2 Methods

### 2.1 Sample

The sample consisted of 48 participants (age 18–33 years, *M* = 23.83, SD = 2.98, 30 female) recruited between February and April 2018. All had Swedish as first language and no prior experience of learning German. Participants received either two movie vouchers or course credit for participation. As an added incentive, participants were informed at the start that the highest scores on cued recall tests would be rewarded with two additional vouchers. Verbal informed consent was obtained from all participants. The sample size was determined based on practical constraints and prior studies using similar VR‐based memory tasks (Hagström and Winman 2018; McLeod 2007).

### 2.2 Materials

#### 2.2.1 Experimental Condition

Sixty German nouns were collected from the frequency word list Wortschatz Deutsch (Universität Leipzig 2016). Words were selected based on regularity and the difficulty of specifying gender, aiming to create a balanced set across the three genders (masculine, feminine and neuter; *der*, *die*, *das*). Two 30‐item lists were constructed, each containing 10 nouns from each gender category. Although the word selection was partly based on subjective judgment, it was also informed by representativeness to ensure comparable difficulty across items.

Two study modes were applied: a 3D application in the experimental condition and paper cards with printed text in the control condition. The 3D VR software was developed in Unity3D with graphics designed using 3DS Max. Display was presented on a computer with a 56.5 × 39.5 cm screen (resolution: 1920 × 1080 pixels, 16:9, refresh rate: 60 hz, frame rate: 60 fps). Participants navigated three virtual environments, one for each of the German gender, connected by a starting area.

**FIGURE 1:**
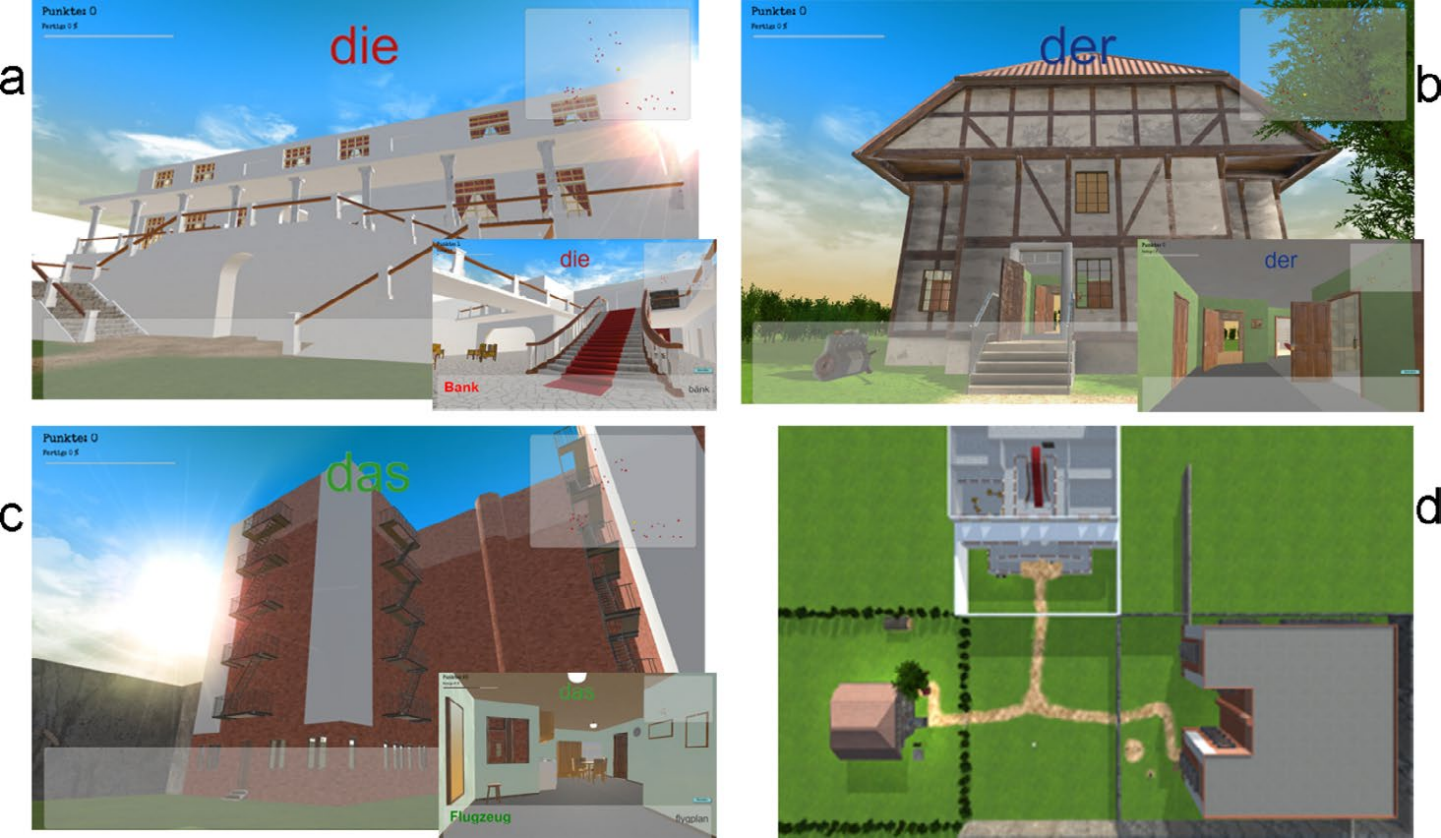
The virtual environments: (a) die; (b) der; (c) das; (d) Floor map of the entire virtual environment.

The *die* environment comprised a white concrete building, while the *das* environment held a brown brick building and the *der* environment a gray half‐timbered building (see Figure 1). Each environment contained ten objects of the assigned gender, visually marked with a green square. When navigating within the die/das/der environments, the corresponding gender was displayed on the upper middle part of the screen. Participants could click on objects to view the German word on the left bottom side of the screen, the Swedish translation on the right bottom side, while the gender was continuously visible at the top middle of the screen. Navigation was controlled using arrow keys. A miniature map indicated the locations of remaining objects.

#### 2.2.2 Control Condition

In the control condition, participants studied 30 paper cards per list, each displaying the German noun with its gender and the Swedish translation above. The order was randomized to match exposure across conditions.

#### 2.2.3 Test Materials

Cued recall tests containing all 60 items were conducted at two time points: an *immediate* test given 30 min after learning (Session 1) and a *delayed* test 3 weeks later (Session 2). The delayed test differed from the immediate test in item order and content, assessing both gender assignment and German word spelling to measure retention across semantic and grammatical dimensions. Since two cued recall tests were utilized on different intervals, test–retest reliability is yielded. The word lists and cued recall tests can be found in the Supporting Information S1.

A free recall test was also administered prior to the cued recall test in Session 2. Free recall performance was assessed in three categories: (a) Swedish words, (b) Gender assignment and (c) German words, and participants rated their confidence for each response on a 1–5 scale.

### 2.3 Data Collection

Motivation, perceived confidence, gaming experience, and demographic variables were collected using online questionnaires (Form I and II). Motivation and perceived efficiency of both study modes were assessed using statements for which participants noted their accordance on a 5‐point Likert scale, where 1 signified no motivation/not at all effective and 5 signified very motivated/very effective.

### 2.4 Procedure

The experiment took place in two sessions, three weeks apart, chosen to balance inherent study limitations with feasibility and a sufficient interval to test memory persistence. The three‐week interval aligns with research on forgetting curves (Ebbinghaus 1913) showing a notable drop in retrieval between 1 week and 31 days (Murre and Dros 2015).

#### 2.4.1 Session 1

Participants were informed about German gender and instructed to learn 60 nouns with their corresponding gender, focusing on gender but also trying to remember the Swedish meaning and the German spelling. Participants were informed that they would be tested in both sessions. In the VR condition, participants received operating instructions and a short description of the idea behind the task. They completed the task twice. In the control condition, 30 paper cards were used. Participants were instructed to look at each card once and not turn any card back or pick up the pile of cards. No time limit was imposed. After a of 30‐min retention interval during which participants completed Form I online (see Table 1) and any remaining time was spent on an unrelated computer‐based task, the immediate cued recall test was administered. The order of these tasks was counterbalanced. Participants were instructed not to actively rehearse the material before Session 2.

**TABLE 1:** Data collection during both sessions.

	Data collection	Measurement (example)
Session 1		
Form I	Game experience	Do you, or have played computer games regularly? (yes/no)
	Game experience level	If yes, specify how much. (Daily, A few times a week, every other week, every other month)
	Perceived efficiency	How efficient would you estimate each learning method to be?
	Motivation	How motivated did you feel when using each learning method?
	Demographic variables	Gender, age
Session 2		
Form II	Perceived efficiency	How efficient would you estimate each learning method to be? (computer game/paper cards)

#### 2.4.2 Session 2

Three weeks later, participants returned for the delayed test session (not necessarily on the exact same time of the day). A free recall test was first administered, where participants had 15 min to write down all remembered Swedish words, corresponding gender, and German words that they could remember. Next, they completed a cued recall test assessing both gender and word spelling. Participants then completed Form II online (see Table 1). All tests were assessed immediately by the first author after the session.

## 3 Results

All statistical tests were two‐tailed with an alpha level of 0.05.

### 3.1 Cued Recall

**FIGURE 2:**
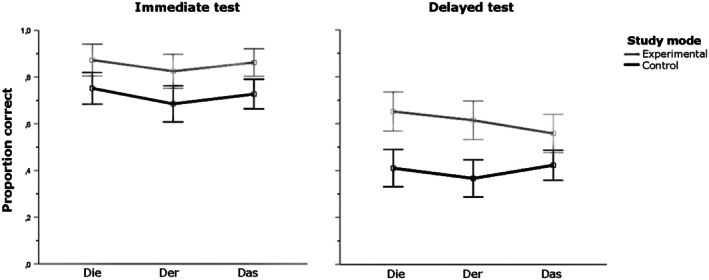
The Interaction between study mode and environment, in both immediate and delayed cued recall memory tests. Vertical error bars indicate 95% confidence intervals.

There were no effects of order of conditions on performance. The main experimental findings appear in Figure 2. As seen, recall differences were in favor of the experimental condition in both tests. A 2 × 3 × 2 repeated measures ANOVA was conducted for immediate and delayed cued gender recall with Study Mode (experimental/control), Gender/Environment, and Retention Interval (Immediate/Delayed) as independent variables.

This analysis showed a main effect of Study Mode, *F*(1, 47) = 41.25, *p* < 0.001, ηp^2^ = 0.47, establishing that cued recall was superior in the experimental study mode. There was a main effect of Retention Interval, *F*(1, 47) = 212.93, *p* < 0.001, ηp^2^ = 0.82 due to substantial forgetting in the delayed test. The main effect of Gender/Environment was significant, *F*(2, 46) = 3.87, *p* = 0.028, ηp^2^ = 0.14, where the *Die* environment produced slightly more correct recall than the other environments. The interaction between Retention Interval and Study Mode was significant, *F*(1, 47) = 8.91, *p* = 0.004, ηp^2^ = 0.16, in that the difference between study modes increased over time. The remaining interactions were all non‐significant (*F*s < 1). Means and standard deviations are found in Table 2.

**TABLE 2:** Mean percentage and standard deviations of correct gender assignment during cued gender assignment recall in the two conditions.

Condition	Cued recall			
	Immediate		Delayed	
	*M*	SD	*M*	SD
Experimental	85.28	18.8	59.93	23.36
Control	71.81	20.7	39.93	22.76

### 3.2 Free Recall

A 3 × 2 ANOVA was conducted on overall Free recall performance, with Type of recall (German word/gender assignment/Swedish word) and Study mode (experimental/control condition) as independent variables. The analysis also revealed a main effect of study mode, *F*(1, 47) = 107.56, *p* < 0.001, ηp^2^ = 0.70; thus, the experimental condition results in better memory retention than the control condition.

**FIGURE 3:**
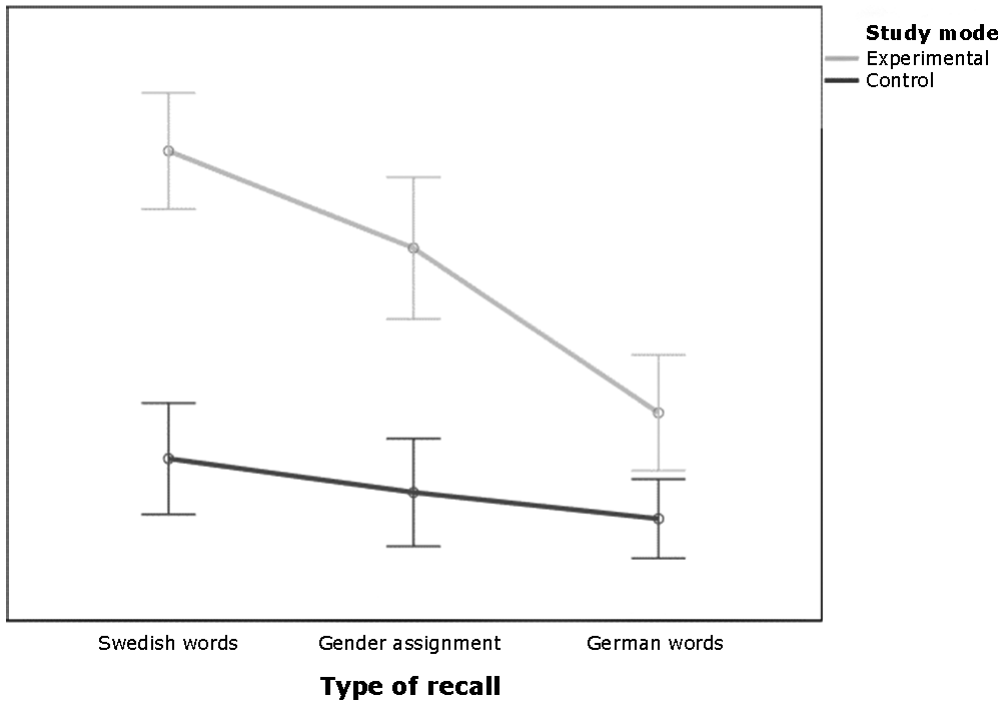
Free recall accuracy of Swedish words, gender assignment and German words. Vertical error bars indicate 95% confidence intervals.

There was a significant Study Mode × Type of Recall interaction, *F*(2, 94) = 32.55, *p* < 0.001, ηp^2^ = 0.41, where the effect of the experimental manipulation was strongest for Swedish words, less strong for gender assignment and weak for German words, as seen in Figure 3. A main effect was found for Type of Recall, *F*(2, 94) = 77.87, *p* < 0.001, ηp^2^ = 0.62, where the strongest recall was produced for Swedish words, followed by gender assignment and lastly, German words.

### 3.3 Potential Moderators

#### 3.3.1 Motivation

A paired samples *t*‐test demonstrated that overall sense of motivation was higher in the experimental condition (*M* = 4.10, SD = 0.93) than in the control condition (*M* = 2.92, SD = 1.15), *t*(46) = 4.78, *p* < 0.001, 95% CI [0.688, 1.687]. Whereas motivation in the control condition is a strong predictor of performance in this condition, motivation does not predict performance in the experimental condition on any measure (see Table 3).

**TABLE 3:** Correlation matrix for motivation ratings of both study modes and cued and free recall performance for both respective conditions (Cont = control, Exp = experimental (MOL) condition) and retention intervals.

	Cued recall		Free recall			
	Immediate	Delayed	Swedish	Gender	German	Overall FR
Motivation						
Cont	0.43**	0.51***	0.51***	0.530***	0.36*	0.50***
Exp	−0.10	0.02	0.21	0.16	0.09	0.16

* *p* < 0.05 (2‐tailed).

** *p* < 0.01 (2‐tailed).

*** *p* < 0.001.

There was a strong negative correlation between motivation ratings for the two conditions (*r*(46) = −0.37, *p* < 0.01). Multiple regression analyses were undertaken with both motivational ratings as predictors and cued recall performance as dependent variables to disentangle these possibly complex effects, see Table 4.

**TABLE 4:** Regression weights (*p*‐values) for multiple regression analyses with self‐rated motivation for the control condition motivation (Cont) and the experimental condition (Exp) as predictors and cued recall performance in the corresponding conditions as dependent variables.

Condition	Motivational predictor				Model *R* ^2^	Model *p*
	Cont		Exp			
	*β*	*p*	*β*	*p*		
Immediate						
Cont	**0.32**	0.02	**−0.29**	0.04	**0.26**	< 0.001
Exp	0.25	0.11	0.00	0.96	0.06	0.22
Delayed						
Cont	**0.48**	0.001	−0.10	0.46	**0.27**	< 0.001
Exp	0.08	0.62	0.15	0.34	0.02	0.63

*Note:* Significant predictors appear in bold text.

This analysis, in correspondence with the zero‐order correlations above, again suggests that motivation predicts performance in both control condition tests, but in neither experimental condition test. In the immediate test, motivation was a significant predictor in both conditions of control performance, experimental condition motivation being a negative predictor. In the delayed test, only control condition motivation predicted the corresponding performance. Thus, motivational factors can only predict learning in the control condition, whereas the experimental condition performance appears to be altogether unrelated to motivation.

#### 3.3.2 Perceived Efficiency

Two paired samples *t*‐tests showed that the participants experienced a higher perceived efficiency of the experimental study mode compared to the control study mode in both sessions (immediate—experimental: *M* = 4.19, SD = 0.87, control: *M* = 2.63, SD = 0.91, *t*(46) = 8.41, *p* < 0.001, [1.189, 1.936], delayed—experimental: *M* = 4.13, SD = 0.64, control: *M* = 2.15, SD = 1.07), *t*(46) = 11.16, *p* < 0.001, 95% CI [1.622, 2.336]. While perceived efficiency of the experimental condition remained similar in both first and second session (*t*(46) = 0.55, *p* = 0.583), perceived efficiency of the control condition had significantly decreased by the second session, *t*(46) = 3.36, *p* = 0.002. No gender differences were found in perceived efficiency for either condition.

Correlations using Pearson's *r* were calculated to explore the relationships between performance in both cued and free recall, motivation, perceived efficacy, and gaming enjoyment.

As shown in Table 5, perceived efficiency correlated mainly with performance for delayed tests in the control condition. Perceived efficiency of the experimental conditions is not predictive of performance.

**TABLE 5:** Correlations between perceived efficiency and cued and free recall performance for both conditions and retention intervals.

Perceived efficiency	Cued recall		Free recall			
	Immediate	Delayed	Swedish	Gender	German	Overall free recall
Immediate						
Cont	0.24	0.26	0.30*	0.30*	0.14	0.27
Exp	−0.02	0.04	−0.12	0.00	0.09	0.00
Delayed						
Cont	0.47**	0.48**	0.53**	0.52**	0.42**	0.52**
Exp	0.06	0.15	0.13	0.19	0.14	0.17

* *p* < 0.05 (2‐tailed).

** *p* < 0.001 (2‐tailed).

#### 3.3.3 Gaming Experience

Correlation analyses were conducted to study relationships between gaming experience, motivation, and performance.

Experienced gamers were faster in the experimental condition, perceived the control condition as less efficient, and reported higher motivation for the experimental and lower motivation for the control condition. As for memory effects, gaming experience was negatively correlated with both cued and free recall in the control condition but not related to experimental condition performance (see Table 6). Because motivation predicted control condition performance, the correlation between gaming experience and this performance could be spurious. In other words, it is possible that gaming experience affects memory performance indirectly through motivation. To test this, gaming experience and control group motivation were both entered in a multiple regression analysis with control group memory performance (cued recall) as the dependent variable. The analysis reveals that both motivation (*β* = 0.38, *p* < 0.001) and gaming experience (*β* = −0.38, *p* < 0.001) remain significant predictors. Thus, the association between gaming experience and memory performance is not merely due to motivation but remains when this variable is controlled for. Gaming experience was negatively related to control condition performance.

**TABLE 6:** Correlations between experience with gaming/experience level, and time on task, perceived efficiency (pooled over retention intervals), motivation, cued (CR) and free recall (FR) performance (pooled over retention intervals) for the control‐ and experimental conditions separately.

	Time		P. eff.		Mot.		CR		FR	
	Cont	Exp.	Cont.	Exp.	Cont.	Exp.	Cont.	Exp.	Cont.	Exp.
Game‐exp	−0.15	−0.57***	−0.37**	0.19	−0.33*	0.42**	−0.50***	−0.03	−0.37**	−0.07
Exp. level	−0.05	−0.48***	−0.32*	0.10	−0.27	0.42**	−0.40**	0.08	−0.31*	0.05

* *p* < 0.05 (2‐tailed).

** *p* < 0.01 (2‐tailed).

*** *p* < 0.001 (2‐tailed).

#### 3.3.4 Biological Gender

Comparisons for both retention intervals with independent samples *t*‐tests with cued recall performance as the dependent variable showed that females outperformed men for words learnt in the control condition, with a difference that approached statistical significance in the immediate test (females: *M* = 76.1%, SD = 20, males: *M* = 64.6%, SD = 20), *t*(46) = 1.91, *p* = 0.06, and reached significance in the delayed test (females: *M* = 44.89%, SD = 21.86%, males: *M* = 31.67%, SD = 22.38%), *t*(46) = 2.01, *p* = 0.05. No gender differences were found in the experimental condition.

The proportion of correct recall from the control condition was subtracted from MOL‐based recall, to render a measure of experimental efficiency. An Independent samples *t‐*test using this efficiency measure in the delayed test as the dependent variable showed that the experimental efficacy was larger for males (*M* = 0.285, SD = 0.210) than for females (*M* = 0.15, SD = 0.19), *t*(46) = 2.30, *p* = 0.026. No gender differences were found for free recall accuracy, *t*(46) = 0.36, *p* = 0.723, 95% CI [−0.653, 0.933]. Males reported higher levels of confidence (*M* = 3.31, SD = 0.77) than females (*M* = 2.77, SD = 0.92), *t*(45) = 2.09, *p* = 0.042, 95% CI [0.203, 1.073], higher levels of gaming experience (males: *M* = 3.61, SD = 1.29, females: *M* = 1.93, SD = 1.51), *t*(46) = 3.93, *p* < 0.001 95% CI [0.819, 2.536], and completed the task in a significantly shorter time (*M* = 0.33 h, SD = 0.13) than women (*M* = 0.46 h, SD = 0.12), *t*(46) = −3.40, *p* = 0.001. An analysis of covariance confirmed that the gender difference in delayed test efficacy was no longer significant after controlling for gaming experience, *F*(1, 45) = 0.21, *p* = 0.65.

An Independent samples *t*‐test revealed that males were also more motivated in the experimental condition (*M* = 4.56, SD = 0.78) than females (*M* = 3.83, SD = 0.91), *t*(46) = 2.79, *p* = 0.008, 95% CI [0.202, 1.243], whereas motivation in the control condition did not differ by gender, *t*(46) = 0.13, *p* = 0.898, 95% CI [−0.650, 0.739].

#### 3.3.5 Time Use

Average completion time was longer in the experimental condition (24.7 min) than in the control condition (15.8 min). To examine whether time‐on‐task accounted for the higher cued and free recall accuracy in the experimental condition, a difference score was computed by subtracting time spent in the control condition from time spent in the experimental condition. The performance in the experimental condition for CR (pooled over both retention intervals) and FR (pooled over the three dependent variables) was subtracted from the corresponding performance in the control condition.

If the superior performance in the experimental condition is due to the longer time spent in this condition, a positive correlation between these variables can be expected. However, these correlations were small and non‐significant (cued recall: *r*(46) = −0.15, *p* = 0.31; free recall: *r*(46) = 0.12, *p* = 0.41), indicating that longer time spent in the experimental condition than in the control condition did not predict larger performance gains. Time spent in the experimental condition alone was negatively correlated with cued recall efficiency (*r*(46) = −0.34, *p* = 0.02), suggesting that longer task duration did not lead to additional benefits. Overall, these results make it unfeasible that the experimental manipulation effects derive from the potential confound in longer time use of the experimental study mode.

## 4 Discussion

### 4.1 Cued and Free Recall: Effect of VR‐Supported MOL

The results of this study demonstrate that cued and free recall of German grammatical gender significantly improved in the VR‐supported MOL condition, persisting on a three‐week interval. Memory effects from the control condition decreased faster, increasing the performance gap over time. These findings replicate and extend earlier research using the same VR 3D application (Hagström and Winman 2018) and a thesis using a MOL approach (McLeod 2007), indicating stable and durable memory representations.

The observed memory benefits from the VR‐supported MOL are also in line with dual‐coding principles (Paivio 2014). By combining spatial context, visual imagery, and semantic association, the VR application may have strengthened these links between item and context, creating multiple retrieval pathways. This may explain why performance in the experimental condition was both higher and more resilient over 3 weeks compared with traditional text‐based learning.

The interaction observed between study mode and retention interval further suggests that VR‐supported MOL influences memory consolidation, not merely initial encoding. Although a three‐week interval does not constitute long‐term memory in the strictest neurocognitive sense, it exceeds the temporal window of short‐term consolidation and allows examination of forgetting dynamics. Compared to the control condition, forgetting in the experimental condition followed a shallower decline, consistent with classical forgetting curves (Ebbinghaus 1913; Murre and Dros 2015) showing that more elaborately encoded material decays more slowly. Thus, the present findings suggest that VR‐supported MOL enhances the durability of memory representations rather than simply boosting immediate recall.

### 4.2 Moderating Factors

Motivation, perceived efficiency, gaming experience, and biological gender were examined as potential moderators of learning. In the control condition, motivation and perceived efficiency predicted performance, and females outperformed males, consistent with well‐established findings in verbal episodic memory (Asperholm et al. 2019). Cognitive‐emotional frameworks (Pekrun et al. 2007) can provide a lens for understanding these patterns, suggesting that traditional text‐based learning relies on affective engagement and effort regulation. When motivation is low, learners may disengage earlier, which could result in weaker encoding. From a metacognitive perspective (Flavell 1979), perceived efficiency is guiding strategy selection, affecting how much effort is invested during the study phase and whether effective encoding strategies are maintained over time. Gaming experience and gender appeared to shape baseline performance, with males' higher gaming experience supporting navigation and efficiency in VR tasks, which may explain why gender differences in memory performance diminished in the experimental condition.

In the VR‐supported MOL condition, the spatial structure and associative cues provided external scaffolding for encoding and retrieval, reducing reliance on motivation and metacognitive effort, and individual differences such as gender disparities. Males gained more than females in this condition, but this difference disappeared when controlling for gaming experience, suggesting that the VR context levels the playing field by offering a more uniform cognitive support.

### 4.3 Practical Implications

The findings have implications for educational practice. VR‐supported mnemonic techniques may be particularly valuable in contexts where learners differ widely in motivation, prior experience, or verbal aptitude, such as language classrooms or adult education settings. By reducing dependence on learner‐specific factors, such tools may promote more equitable learning outcomes. While the MOL typically requires preparation and training, VR applications can facilitate visual encoding in a more accessible manner. In practical terms, VR‐supported MOL could be implemented as a complementary educational strategy for vocabulary acquisition, grammar learning, or other domains requiring durable associative memory. Importantly, the observed benefits appear to reflect underlying cognitive mechanisms that support retention beyond immediate recall, though the durability of these effects over longer intervals should be further examined.

### 4.4 Limitations

The study has several limitations. First, the sample was relatively small and most participants were university students in their mid‐twenties, likely familiar with technological tools. Second, a formal power analysis was not conducted, and the sample size was determined based on practical constraints and prior similar studies, which may limit the generalizability and robustness of the findings. Third, the experimental condition combined the use of MOL and VR, rather than separating the two into multiple conditions, preventing disentanglement of the individual contributions of each method. Future studies could utilize factorial designs to isolate these effects, by testing the effects of the MOL and VR separately as well as together to see which one contributes to memory improvements. Fourth, distributing a test immediately after memorization in the first session may have caused test effects, where the act of testing itself enhances learning (Roediger III et al. 2011). However, the immediate test was necessary to compare short‐ and long‐term memorization. Finally, the retention interval was extended from 1 week in our previous study to 3 weeks in the present study. Future research with more extensive resources is warranted to examine learning effects on longer retention intervals.

## 5 Conclusion

Expanding our knowledge on virtual learning environments, the main finding of this experiment was that memory recall of German gender assignment persisted after a three‐week retention interval, suggesting long‐term efficiency of harnessed memory. In addition, stronger motivation towards virtual environments was not reflected in recall, which indicates that this efficiency is supported by other, possibly automatically induced cognitive factors related to visualization processes. A further pursuit of investigation of psychological variables relevant for virtual learning environments and applied implications for educational settings is of importance.

## Author Contributions

Both authors substantially contributed to the conception and design of the work, as well as the writing of the manuscript. J.H. collected the data, created the visualizations, and wrote the initial draft.

## Funding

The authors have nothing to report.

## Ethics Statement

The authors have nothing to report.

## Consent

Verbal informed consent was obtained from all participants.

## Conflicts of Interest

The authors declare no conflicts of interest.

## Supporting information


**Appendix S1:** The two 30‐item word lists, each containing 10 nouns from each of gender category.

**Appendix S2:** The immediate cued recall test containing all 60 items conducted 30 min after learning (Session 1).

**Appendix S3:** The delayed cued recall test 3 weeks after learning (Session 2).

**Appendix S4:** The free recall test, administered prior to the cued recall test (Session 2).

## Data Availability

The data that support the findings of this study are available from the corresponding author upon reasonable request.
