# Supplementary material for: Playing for Keeps: Long‐Term Recall With an Application Using Virtual Reality and the Method of Loci
Source: Scand J Psychol. 2026 Mar 23;67(4):1090–8. doi: 10.1111/sjop.70089 (PMC13352563; doi:10.1111/sjop.70089)
Supplement: Supplementary file 1 — Appendix S1: The two 30‐item word lists, each containing 10 nouns from each of gender category. [file SJOP-67-1090-s002.pdf]

Appendix A

Word list 1 and 2

This appendix consists of the two lists of Germans nouns, their determinative and Swedish translation, used as learning material in the experimental and control condition.

List 1

Swedish translation	Determinative	German word
Bänken	Die	Bank
Säkringen	Die	Sicherung
Klotet	Die	Kügel
Formen	Die	Form
Klämman	Die	Klammer
Tunnan	Die	Tonne
Metspöt	Die	Angel
Muren	Die	Mauer
Gaffeln	Die	Gabel
Diskotek	Die	Diskothek
Hindret	Das	Hindernis
Sängen	Das	Bett
Järnet	Das	Eisen
Astman	Das	Asthma
Geväret	Das	Gewehr
Landet	Das	Land
Kilot	Das	Kilo
Ägget	Das	Ei
Spelet	Das	Spiel
Soffan	Das	Sofa
Ståndet	Der	Stand
Motorn	Der	Motor
Vinet	Der	Wein
Soporna	Der	Müll
Kaffet	Der	Kaffee
Paraplyet	Der	Schirm
Spegeln	Der	Spiegel
Draperiet	Der	Vorhang
Brottet	Der	Bruch
Teven	Der	Fernseher

List 2

**Swedish translation**	**Determinative**	**German word**
Mjölken	Die	Milch
Valet	Die	Wahl
Brandkåren	Die	Feuerwehr
Gåvan	Die	Gabe
Skiktet	Die	Schicht
Tiden	Die	Zeit
Vykortet	Die	Ansicht
Ammunitionen	Die	Munition
Musen	Die	Maus
Trafikljuset	Die	Ampel
Kabeln	Das	Kabel
Glassen	Das	Eis
Målet	Das	Ziel
Lampan	Das	Licht
Bilen	Das	Auto
Affishen	Das	Plakat
Halsduken	Das	Halstuch
Linjalen	Das	Lineal
Akvariet	Das	Aquarium
Flygplanet	Das	Flugzeug
Carporten	Der	Carport
Grunden	Der	Grund
Bordet	Der	Tisch
Greppet	Der	Griff
Avloppet	Der	Ablauf
Osten	Der	Käse
Buren	Der	Käfig
Bilstolen	Der	Autositz
Bollen	Der	Ball
Skon	Der	Schuh
