# Supplementary material for: Playing for Keeps: Long‐Term Recall With an Application Using Virtual Reality and the Method of Loci
Source: Scand J Psychol. 2026 Mar 23;67(4):1090–8. doi: 10.1111/sjop.70089 (PMC13352563; doi:10.1111/sjop.70089)
Supplement: Supplementary file 2 — Appendix S2: The immediate cued recall test containing all 60 items conducted 30 min after learning (Session 1). [file SJOP-67-1090-s001.pdf]

Appendix B

Cued recall test 1

This appendix consists of the Cued recall test conducted during Session 1.

Var vänlig fyll i rätt bestämd artikel (der, die eller das) i den tomma mittenraden.

#	**Swedish translation**	**Determinative**	**German word**
1	Akvariet		Aquarium
2	Halsduken		Halstuch
3	Bordet		Tisch
4	Gaffeln		Gabel
5	Spegeln		Spiegel
6	Geväret		Gewehr
7	Bilstolen		Autositz
8	Brottet		Bruch
9	Skiktet		Schicht
10	Järnet		Eisen
11	Bänken		Bank
12	Draperiet		Vorhang
13	Formen		Form
14	Klotet		Kügel
15	Glassen		Eis
16	Vykortet		Ansicht
17	Bollen		Ball
18	Teven		Fernseher
19	Avloppet		Ablauf
20	Brandkåren		Feuerwehr
21	Affischen		Plakat
22	Skon		Schuh
23	Spelet		Spiel
24	Musen		Maus
25	Motorn		Motor
26	Kaffet		Kaffee
27	Målet		Ziel
28	Landet		Land
29	Metspöt		Angel
30	Valet		Wahl

Fortsätt på andra sidan ➔

31	Carporten		Carport
32	Grunden		Grund
33	Soffan		Sofa
34	Greppet		Griff
35	Osten		Käse
36	Ammunitionen		Munition
37	Buren		Käfig
38	Tiden		Zeit
39	Ståndet		Stand
40	Säkringen		Sicherung
41	Kabeln		Kabel
42	Astman		Asthma
43	Soporna		Müll
44	Flygplanet		Flugzeug
45	Trafikljuset		Ampel
46	Diskotek		Diskothek
47	Mjölken		Milch
48	Kilot		Kilo
49	Hindret		Hindernis
50	Tunnan		Tonne
51	Gåvan		Gabe
52	Sängen		Bett
53	Klämman		Klammer
54	Lampan		Licht
55	Linjalen		Lineal
56	Muren		Mauer
57	Bilen		Auto
58	Paraplyet		Schirm
59	Ägget		Ei
60	Vinet		Wein
