# Supplementary material for: Playing for Keeps: Long‐Term Recall With an Application Using Virtual Reality and the Method of Loci
Source: Scand J Psychol. 2026 Mar 23;67(4):1090–8. doi: 10.1111/sjop.70089 (PMC13352563; doi:10.1111/sjop.70089)
Supplement: Supplementary file 3 — Appendix S3: The delayed cued recall test 3 weeks after learning (Session 2). [file SJOP-67-1090-s003.pdf]

Appendix C

Cued recall test 2

This appendix consists of the Cued recall test conducted during Session 2.

Var vänlig fyll i rätt bestämd artikel (der/die/das) i mittenkolumnen, samt tyskt ord i kolumnen till höger.

#	**Swedish translation**	**Determinative**	**German word**
1	Kilot		
2	Ammunitionen		
3	Bordet		
4	Gaffeln		
5	Carporten		
6	Brandkåren		
7	Bilstolen		
8	Formen		
9	Spelet		
10	Järnet		
11	Paraplyet		
12	Geväret		
13	Målet		
14	Halsduken		
15	Glassen		
16	Musen		
17	Bollen		
18	Linjalen		
19	Grunden		
20	Bilen		
21	Valet		
22	Bänken		
23	Mjölken		
24	Diskotek		
25	Motorn		
26	Spegeln		
27	Kabeln		
28	Teven		
29	Skiktet		
30	Brottet		

Fortsätt på andra sidan ➔

31	Metspöt		
32	Avloppet		
33	Soffan		
34	Greppet		
35	Affischen		
36	Kaffet		
37	Buren		
38	Tiden		
39	Ståndet		
40	Säkringen		
41	Skon		
42	Osten		
43	Soporna		
44	Flygplanet		
45	Trafikljuset		
46	Vinet		
47	Akvariet		
48	Vykortet		
49	Hindret		
50	Tunnan		
51	Ägget		
52	Astman		
53	Klämman		
54	Lampan		
55	Draperiet		
56	Muren		
57	Klotet		
58	Sängen		
59	Landet		
60	Gåvan		
