# Supplementary material for: Playing for Keeps: Long‐Term Recall With an Application Using Virtual Reality and the Method of Loci
Source: Scand J Psychol. 2026 Mar 23;67(4):1090–8. doi: 10.1111/sjop.70089 (PMC13352563; doi:10.1111/sjop.70089)
Supplement: Supplementary file 4 — Appendix S4: The free recall test, administered prior to the cued recall test (Session 2). [file SJOP-67-1090-s004.pdf]

Appendix D

Free recall test

This appendix consists of the Free recall test conducted during Session 2.

Fyll i de svenska ord, artiklar och tyska ord du minns från tillfälle 1. Uppskatta din säkerhet (1= osäker, 5= mycket säker).

#	**Swedish translation**	**Determinative**	**German word**	**Level of Certainty**				
1				1	2	3	4	5
2				1	2	3	4	5
3				1	2	3	4	5
4				1	2	3	4	5
5				1	2	3	4	5
6				1	2	3	4	5
7				1	2	3	4	5
8				1	2	3	4	5
9				1	2	3	4	5
10				1	2	3	4	5
11				1	2	3	4	5
12				1	2	3	4	5
13				1	2	3	4	5
14				1	2	3	4	5
15				1	2	3	4	5
16				1	2	3	4	5
17				1	2	3	4	5
18				1	2	3	4	5
19				1	2	3	4	5
20				1	2	3	4	5
21				1	2	3	4	5
22				1	2	3	4	5
23				1	2	3	4	5
24				1	2	3	4	5
25				1	2	3	4	5
26				1	2	3	4	5
27				1	2	3	4	5
28				1	2	3	4	5
29				1	2	3	4	5
30				1	2	3	4	5

Fortsätt på andra sidan ➔

31				1	2	3	4	5
32				1	2	3	4	5
33				1	2	3	4	5
34				1	2	3	4	5
35				1	2	3	4	5
36				1	2	3	4	5
37				1	2	3	4	5
38				1	2	3	4	5
39				1	2	3	4	5
40				1	2	3	4	5
41				1	2	3	4	5
42				1	2	3	4	5
43				1	2	3	4	5
44				1	2	3	4	5
45				1	2	3	4	5
46				1	2	3	4	5
47				1	2	3	4	5
48				1	2	3	4	5
49				1	2	3	4	5
50				1	2	3	4	5
51				1	2	3	4	5
52				1	2	3	4	5
53				1	2	3	4	5
54				1	2	3	4	5
55				1	2	3	4	5
56				1	2	3	4	5
57				1	2	3	4	5
58				1	2	3	4	5
59				1	2	3	4	5
60				1	2	3	4	5
